# Patient satisfaction is biased by renovations to the interior of a primary care office: a pretest-posttest assessment

**DOI:** 10.1186/s12913-016-1647-4

**Published:** 2016-08-11

**Authors:** Raphaël Tièche, Bruno R. da Costa, Sven Streit

**Affiliations:** 1 Primary Care Office, Grenchen, Switzerland; 2 Institute of Primary Health Care (BIHAM), University of Bern, Gesellschaftsstrasse 49, 3012 Bern, Switzerland

**Keywords:** Patient satisfaction, Primary care, Quality of care, and Change of appearance

## Abstract

**Background:**

Measuring quality of care is essential to improve primary care. Quality of primary care for patients is usually assessed by patient satisfaction questionnaires. However, patients may not be able to judge quality of care without also reflecting their perception of the environment. We determined the effect that redesigning a primary care office had on patient satisfaction. We hypothesized that renovating the interior would make patients more satisfied with the quality of medical care.

**Methods:**

We performed a Pretest-Posttest analysis in a recently renovated single-practice primary care office in Grenchen, Switzerland. Before and after renovation, we distributed a questionnaire to assess patient satisfaction in four domains. We chose a Likert scale (1 = very poor to 6 = very good), and 12 quality indicators, and included two consecutive samples of patients presenting at the primary care office before (*n* = 153) and after (*n* = 153) interior design renovation.

**Results:**

Response rate was high (overall 85 %). The sample was similar to the enlisted patient collective, but the sample population was older (60 years) than the collective (52 years). Patient satisfaction was higher for all domains after the office was renovated (*p* < 0.01–0.001). Results did not change when we included potential confounders in the multivariable model (*p* < 0.01).

**Conclusions:**

Renovating the interior of a primary care office was associated with improved patient satisfaction, including satisfaction in domains otherwise unchanged. Physician skills and patient satisfaction sometimes depend on surrounding factors that may bias the ability of patients to assess the quality of medical care. These biases should be taken into account when quality assessment instruments are designed for patients.

**Electronic supplementary material:**

The online version of this article (doi:10.1186/s12913-016-1647-4) contains supplementary material, which is available to authorized users.

## Background

Measuring quality of care is essential to improve primary care, but measuring quality of care is difficult and there is, as yet, no established method to effectively assess quality [1]. We cannot know what to change unless we know what is wrong. In Switzerland, the concept of quality in primary care is being elaborated by a task force established by the occupational union (MFE) of general practitioners (GPs) [2]. The concept includes four topics: quality circles (working groups of GPs), patients, health care providers, and the next generation of GPs.

Quality of primary care for patients is usually assessed by patient questionnaires and comparative benchmarks of scores for GP offices, but questionnaires are designed to measure the patient’s subjective perception of quality. Conclusions based solely on subjective quality of care assessments by patients may bias results, and limit their usefulness as benchmarks for primary care offices. For example, a recent study shows how difficult it is to separate the role of the physical environment from the effect of social forces on patient wellbeing [3].

An earlier UK study found that upgrading the primary care environment can increase patient satisfaction [4]. This and similar studies found that environmental upgrades also improve patient perceptions about the health care they receive [5, 6]. Patients may not be able to judge quality of care without also reflecting their perception of the environment. On the other hand, Gosling et al. conducted two studies that suggest that observer impressions are often accurate and rely on valid environmental clues to correctly judge the characteristics of staff and other room occupants [7]. Since patients are usually not well-informed about standards of practice, and because they do not have a medical education, they often judge a doctor’s performance based on the personality of the physician, and the physical environment [8]. Patients may believe that a redesign reflects the provider’s desire to care for their wellbeing, and may then assume that the provider puts the same energy into providing medical services. This may be why attractive and comfortable waiting rooms, and good lighting can result in higher quality of care assessments from patients [9]. Patient assessment of quality of care may not describe accurately the standards of the medical practitioners who treat them.

We hypothesized that upgrading the interior design of an office would improve patient perceptions of quality of care, absent any other changes. We tested our hypothesis with pre- and post-renovation questionnaires to measure the amount that renovating a GP office changed patient satisfaction in other dimensions, like quality of treatment or physician reliability.

## Methods

### Study design

This is an observational study with a pretest-posttest assessment design.

### Setting

Our study was conducted in the group practice of two GPs in Grenchen, a city with 17,000 inhabitants, located in the countryside in the northwestern part of Switzerland. The GP office was founded in 1981 by the senior GP; the junior GP joined the practice in 2013. Patients in Switzerland are free to choose their GP. Two out of three patients at office have seen their physicians there for more than 5 years. The patients who visited the office within the last 2 years were mostly women (53 %); mean age was 52 years. Most patients (80 %) speak German.

### Population and intervention

**Fig. 1:**
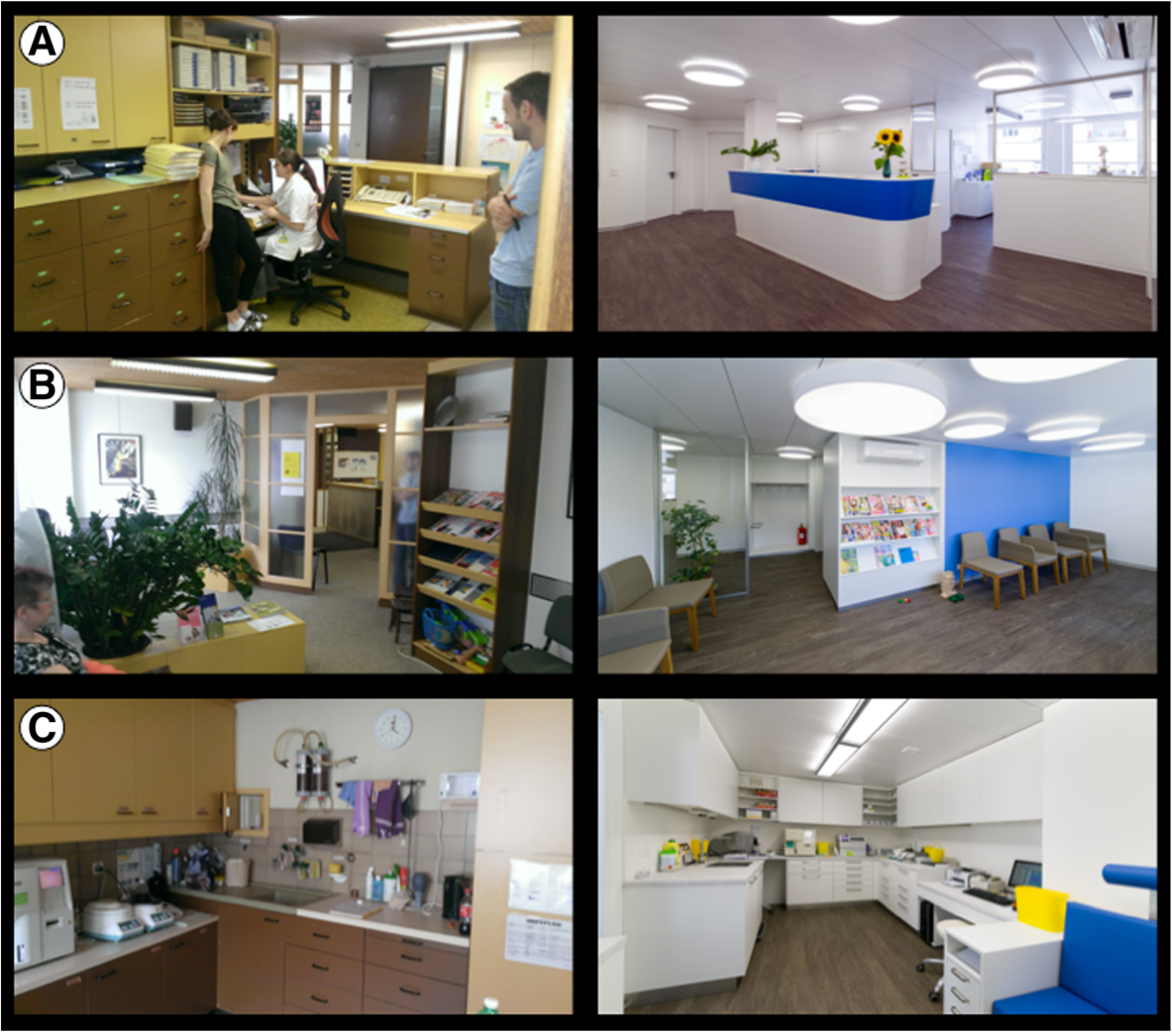
Panels **a**, **b**, and **c** display the GP office before (left side) and after renovation (right side). **a** reception, **b** waiting room, **c** laboratory. We received consent to publish the pictures from all people on (**a**)

For 6 weeks, from June 23 until August 8, 2014, the practice was renovated. The entry hall, the reception area, the laboratory where patients give blood, the waiting room, the staff room, and the pharmacy were refurnished (Fig. 1). The consultation rooms were not changed. No new working processes or instruments were introduced during the observation period. Both GPs continued working the same number of days before and after renovation (senior GP 3 days/week, junior GP 4 days/week). A medical assistant who had been working 3 days/week left the team after the renovation; four medical assistants remained (3 working full time, 1 working 2.5 days/week). We performed a 1-week pilot study to assess feasibility and reply rate before the renovations began.

On April 7, 2014, while patients were waiting to consult the doctor, a medical assistant assured patients of anonymity and asked for oral consent before she distributed survey questionnaires (Additional file 1). The GPs did not distribute the questionnaire or refer to it during consultation. The questionnaire was offered to all German-speaking patients aged >18 years, in order of arrival, until all 180 questionnaires were handed out. Patients could fill out the forms before or after they saw the doctor. After the consultation, and before they left the office, a medical assistant asked patients if they had completed the questionnaire and collected them.

The week after renovation, a medical assistant distributed another 180 questionnaires, using the same strategy as for the first. The second questionnaire was the same as the first, but was printed on yellow paper, so patients who had filled it out in the first round would not think they were being given an identical questionnaire. Patients were told this questionnaire would be for post-assessment after renovation and to fill out the questionnaire only if they had already attended the office prior to renovation (>2 months). For post-assessment patients attending the office for <2 months, this was an exclusion criteria.

To replicate the sample size of similar surveys done by two Swiss institutes [10, 11], we decided to recruit at least 150 patients for each of the pre- and post-renovation assessments. Based on the pilot assessment, we expected an 80 % or greater response rate, so we handed out 180 survey questionnaires during pre- and post-assessments.

Swiss law on human research (Humanforschungsgesetz, HFG) does not require ethics committee approve to collect and analyze anonymous non-medical data.

### Measuring patient satisfaction

We assessed patient satisfaction by asking patients to grade 12 quality indicators on a Likert scale from 1 (very poor) to 6 (very good). We assessed the following characteristics: 1) appearance of the facility; 2) condition of diagnostic equipment; 3) level of hygiene at the practice; 4) punctuality and dependability of the staff; 5) promptness of staff response to patient needs; 6) dress and grooming of the medical assistants; 7) friendliness and courtesy of the medical assistants; 8) attentiveness and responsiveness of the GP to patient needs; 9) GP’s perceived level of expertise; 10) GP’s level of empathy; 11) overall medical performance of the office; and, 12) overall patient satisfaction with the office (Table 1). We chose these indicators after we performed a literature search to bring our work into accord with published studies [10] and in line with two questionnaires already commonly used to assess patient satisfaction in Switzerland [11, 12]. To interpret the results, we grouped the 12 quality indicators into four domains, each of which covers a specific topic, and then averaged the corresponding items: 1) appearance of the office; 2) qualities of the medical assistants; 3) qualities of the GPs; and, 4) general satisfaction. We used Cronbach’s alpha to assess whether items within each of the four groups of quality indicators seemed to measure the same construct (Cronbach’s alpha was good, ranging from 0.75 to 0.88).

**Table 1:** Domains of quality indicators selected to measure patient satisfaction

Domains	Quality indicators	
Appearance of the office	1	Appearance of the facility
	2	Diagnostic equipment
	3	Level of hygiene
	4	Punctuality and dependability
	5	Prompt response to patient needs
Qualities of the medical assistant	6	Dress and grooming of the medical assistants
	7	Friendliness and courtesy of the medical assistants
Qualities of the general practitioner	8	GP is attentive and responsive to patient’s needs
	9	GP’s level of expertise
	10	GP’s level of empathy
General satisfaction	11	Medical performance of the office
	12	Overall satisfaction with the office

### Statistical analysis

We used means and standard deviations, or numbers and proportions, for descriptive statistics. We calculated means and 95 % confidence intervals of pre- and post-assessments for each of the four domains, and then compared them, using linear regression with robust standard errors, so we could account for the possibility some patients were included in both phases of the assessment. We then created multivariable models to control for the potential influence of confounders by simultaneously including the following variables in the model: age, sex, GP assignment, and, duration of assignment. We used STATA release 13.1 for all analyses (Stata Corp, College Station, TX, USA). A *p*-value of <0.05 was considered statistical significant.

## Results

### Baseline characteristics

A total of 153 (85 %) patients participated in the pre-renovation survey; the same number participated in the post-renovation survey (Table 2). We excluded 16 patients in the post-assessment phase because they had attended the office for <2 months and had therefore not seen the office prior to renovation. The overall mean age of the study population was 60 ± 18 years (SD); of these, 53 % were women. Most patients were seen by the senior GP (53 %), and most patients had attended the practice for over 5 years (67 %). The patients we included were, on average, about 8 years older than the average of all patients who attended the practice (60 years vs. 52 years), probably because we included only patients older than 18.

**Table 2:** Basic characteristics of patients before and after renovation

Characteristics^a^	Before renovation (*n* = 153)	After renovation (*n* = 153)	*P*-value
Patients			
Age, years (SD)	60.9 (18)	58.6 (18)	0.25
	*n* = 149	*n* = 151	
Women, n (%)	78 (51)	85 (55)	0.56
	*n* = 149	*n* = 151	
GP assignment, n (%)			0.77
senior GP	81 (53)	79 (52)	
junior GP	46 (30)	47 (31)	
both	18 (12)	22 (14)	
	*n* = 145	*n* = 148	
Duration of assignment, n (%)			0.005
< 2 months	12 (8)	0 (0)	
< 1 year	17 (11)	27 (18)	
1–5 years	21 (14)	18 (12)	
> 5 years	101 (66)	105 (69)	
	*n* = 151	*n* = 150	

^a^ due to missing data the n of each group is listed beneath each item

The groups of patients who filled out the questionnaire before and after renovation were similar in age, sex, and assignment to physicians. The statistical difference in length of assignment to the office resulted from excluding patients who attended for <2 months in the post-renovation phase (Table 2).

### Patient satisfaction

**Fig. 2:**
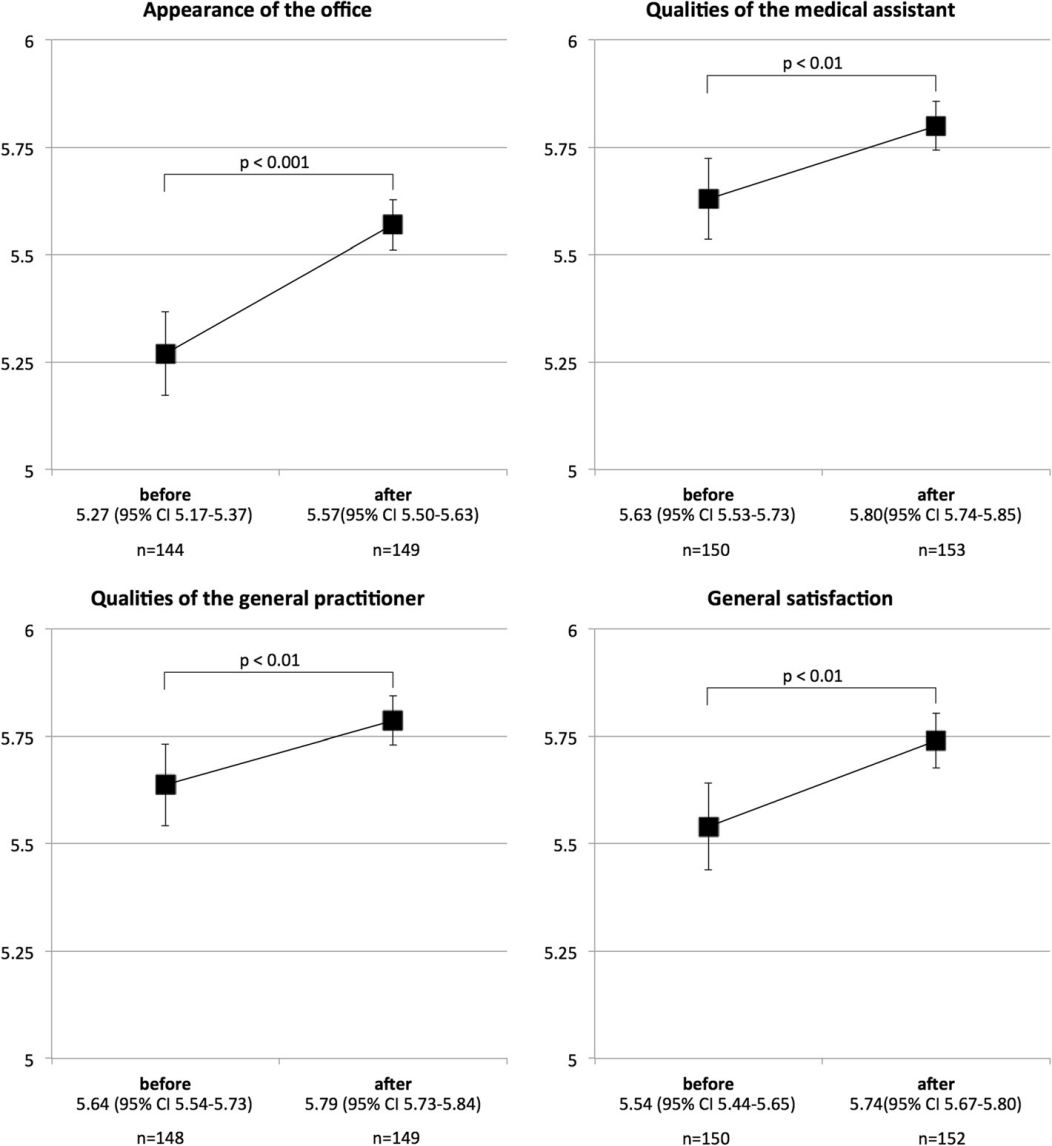
Patient satisfaction per domain before and after renovation

Correlations within the four domains were concorded with a Cronbach’s alpha between 0.75 and 0.88. Most patients thought the quality of care before renovation was high or very high; most patients graded the characteristics between 5 and 6 in all domains. Figure 2 displays the number of patients and mean values per domain, before and after renovation. Most patients filled out all items on the questionnaire. The lowest response rate (96 %) was for the domain “appearance of the office,” and the highest (99 %) was for the domain “qualities of the medical assistant”. Mean values before renovation were similar across all domains. The domain “appearance of the office” had the lowest mean value (5.27; 95 % confidence interval 5.17 to 5.37). Mean value for all domains increased a statistically significant amount after renovation (*p* < 0.01). The largest improvement in patient satisfaction was in the domain “appearance of the office” (*p* < 0.001), though the magnitude of improvement was similar across all domains. For all domains, results were unchanged after we included potential confounders in the multivariable model (Table 3).

**Table 3:** Comparison of patient satisfaction before and after renovation using univariate and multivariate regression models

Domains	Change in satisfaction^a^ after renovation in 4 domains			
	Univariate (95 % CI)	*P*-value	Multivariate^b^ (95 % CI)	*P*-value
Appearance of the office	0.29 (0.17–0.41)	<0.001	0.26 (0.14–0.38)	<0.001
Qualities medical assistant	0.16 (0.06–0.28)	0.003	0.17 (0.05–0.28)	0.006
Qualities GP	0.15 (0.04–0.26)	0.008	0.15 (0.04–0.27)	0.01
General satisfaction	0.20 (0.08–0.32)	0.001	0.17 (0.05–0.29)	0.006

^a^on a scale from 1 (unacceptable) to 6 (very good), thus e.g. 0.26 means an increase by 0.26 on that scale after renovation

^b^adjusted for age, sex, GP assignment, and duration of assignment

## Discussion

Patient satisfaction significantly increased in all domains after the renovation, even though only exterior quality indicators changed. Patient satisfaction with the appearance of the office improved, but so did their satisfaction with office and medical processes, patient management, and other measures like the dress and grooming of medical assistants, and the level of expertise of the GP.

Ulrich RS et al., long ago recognized the impact of visual change on patient satisfaction [13]. In Ulrich’s study of hospitalized patients after cholecystectomy in Pennsylvania, USA, those patients assigned to a room with a view of nature were less likely to make negative comments on evaluations. Likewise, Otani K et al. [14] examined patients in a primary care setting in the US and found that patients looked for surrogate indicators for quality of physician care among the measures available to them. DeLia D et al. [15] interviewed outpatients in New York, USA, to determine which aspects of the ambulatory care visit had the greatest influence on patients’ overall site evaluation. In addition to the personal interaction between patients and physicians, and provider continuity, the appearance of the facility was a high priority, which may explain why, in our study, patient perception of quality of care increased in all domains after the clinic was renovated.

### Limitations and strengths

Our study has several limitations and some strengths. Our questionnaire was not validated, but the questions we asked covered the same domains as common questionnaires in Switzerland [10, 11]. We distributed our questionnaire consecutively but they did not include a uniform identification number, so we could not determine if there was overlap between patients in the pre- and post-assessments. Instead, we used robust regression to account for dependency between the two samples. Our questionnaire was in German, so it self-excluded patients without sufficient German language skills, but 80 % of all enlisted patients were German speakers. Because we included only patients >18 years old, the group we sampled was older than the general patient population of the GP office. Our results can thus only be generalized to patients >18 years. We did not seek information about patient social class, housing tenure, or amount of education, though these may be related to patient satisfaction [16, 17]. Quality measurement depends on many factors. We attempted to limit those factors by changing only the interior of the clinic, and leaving other factors, including staff and processes, intact. We included a sample of patients similar in size to that used by Swiss governmental and non-government institutions to measure patient satisfaction.

Our findings suggest that caution is indicated when interpreting subjective patient satisfaction questionnaires in primary care because patient satisfaction is not objective, and depends on factors beyond simple medical care. Changing the patient’s environment can have a very strong effect on a patient’s assessment of other domains of quality, biasing the patient’s response. Patient questionnaires do evaluate overall patient satisfaction with a GP office at a particular point in time, and can be structured to provide patients with the opportunity to suggest improvements. However, it would be useful to adjust for factors like time since last renovation when interpreting their results, especially if they are used to set and compare benchmarks for GP offices.

Future research should determine if the increase in patient satisfaction after renovation is only a temporary “bounce”, in which case we might see satisfaction drop back to pre-renovation levels over time. It would also be useful to know if patient increase in satisfaction with quality of care is also associated with a real improvement in a non-subjective quality of care indicator and if higher satisfaction is more evident in offices with lower initial scores on attractiveness than in offices already considered attractive.

## Conclusions

Patient perceptions of the skills of physicians, and patient satisfaction with topics in different domains depend strongly on outside factors, like renovation, that can bias patient assessment. Our study is in line with the body of research that indicates that improving the interior of a GP office also improves overall patient satisfaction, including in domains where nothing changed, creating biases that must be accounted for when researchers design and analyze questionnaires about quality of care.

## Additional file

Additional file 1: Survey. (DOCX 131 kb)

## Abbreviations

CI: Confidence Interval
GP: General Practitioners
MFE: Swiss Occupational Union of General Practitioners and Pediatricans
SD: Standard Deviation
