# Supplementary material for: Patient satisfaction is biased by renovations to the interior of a primary care office: a pretest-posttest assessment
Source: BMC Health Serv Res. 2016 Aug 11;16:373. doi: 10.1186/s12913-016-1647-4 (PMC4982145; doi:10.1186/s12913-016-1647-4)
Supplement: Additional file 1: — Survey. (DOCX 131 kb) [file 12913_2016_1647_MOESM1_ESM.docx]

**Additional file 1: Survey**

**Survey on quality of the GP office**

**Thank you for your participation! We will analyse your replies anonymously.**

Date: _______________ Year of birth: _______ Sex: female male

Tick the box of your GP assignment: Dr. Tièche junior

Dr. Tièche senior

Duration of assignment: < 2 months

< 1 year

1-5 years

>5 years

**Please rate the items below using these marks:**

1 = inacceptable 2 = highly insufficent 3 = insufficient

4 = satifactory 5= good 6 = very good

**Appearance of the office**

1. Appearance of the facility

1

2

3

4

5

6

1. Diagnostic equipment

1

2

3

4

5

6

1. Level of hygiene

1

2

3

4

5

6

1. Punctuality and dependability

1

2

3

4

5

6

1. Prompt response to patient needs

1

2

3

4

5

6

**Qualities of the medical assistant**

1. Dress and grooming of the medical assistants

1

2

3

4

5

6

1. Friendliness and courtesy of the medical assistants

1

2

3

4

5

6

**Qualities of the general practitioner (GP)**

1. GP is attentive and responsive to patient’s needs

1

2

3

4

5

6

1. GP’s level of expertise

1

2

3

4

5

6

1. GP’s level of empathy

1

2

3

4

5

6

**General satisfaction**

1. Medical performance of the office

1

2

3

4

5

6

1. Overall satisfaction with the office

1

2

3

4

5

6

**General remarks**

We thank you for your participation
